# Highly Elastic and Water Stable Zein Microfibers as a Potential Drug Delivery System for Wound Healing

**DOI:** 10.3390/pharmaceutics12050458

**Published:** 2020-05-18

**Authors:** Alma Akhmetova, Georg-Marten Lanno, Karin Kogermann, Martin Malmsten, Thomas Rades, Andrea Heinz

**Affiliations:** 1 LEO Foundation Center for Cutaneous Drug Delivery, Department of Pharmacy, University of Copenhagen, 2100 Copenhagen, Denmark; alma.akhmetova@sund.ku.dk (A.A.); martin.malmsten@sund.ku.dk (M.M.); thomas.rades@sund.ku.dk (T.R.); 2 Institute of Technology, University of Tartu, 50411 Tartu, Estonia; georg.lanno@gmail.com; 3 Institute of Pharmacy, University of Tartu, 50411 Tartu, Estonia; karin.kogermann@ut.ee; 4 Department of Physical Chemistry, Lund University, 221 00 Lund, Sweden

**Keywords:** biomaterial, electrospinning, PEO, stearic acid, tetracycline hydrochloride

## Abstract

The development of biomaterials for wound healing applications requires providing a number of properties, such as antimicrobial action, facilitation of cell proliferation, biocompatibility and biodegradability. The aim of the present study was to investigate morphological and mechanical properties of zein-based microfibers, ultimately aimed at creating an environment suitable for wound healing. This was achieved through co-axial electrospinning of core–shell microfibers, with zein protein in the core and polyethylene oxide (PEO) in the shell. Small amounts of PEO or stearic acid were additionally incorporated into the fiber core to modify the morphology and mechanical properties of zein fibers. The presence of PEO in the core was found to be essential for the formation of tubular fibers, whereas PEO in the shell enhanced the stability of the microfibers in water and ensured high elasticity of the microfiber mats. Tetracycline hydrochloride was present in an amorphous form within the fibers, and displayed a burst release as a result of pore-formation in the fibers. The developed systems exhibited antimicrobial activity against *Staphylococcus aureus* and *Escherichia coli*, and showed no cytotoxic effect on fibroblasts. Biocompatibility, antimicrobial activity and favorable morphological and mechanical properties make the developed zein-based microfibers a potential biomaterial for wound healing purposes.

## 1. Introduction

With the increase of the aging population, as well as related chronic health conditions such as diabetes, the incidence of non-healing chronic wounds is on the rise [1]. Specifically, complications associated with chronic wounds include bacterial infection, tissue necrosis and subsequent amputations. In extreme cases, mortality rates similar to or even higher than those for some cancer types have been reported [2]. Expensive long-term treatments of such wounds place a considerable burden on patients and the healthcare system, including not only physiological measures, but also psychological support, due to the lowered quality of life and increased incidence in depression [1,3]. Considerable efforts have been directed towards facilitating wound healing outcomes by developing biomaterials that allow oxygen exchange, absorb exudate, and provide mechanical protection from further injuries [4,5]. Among different types of biomaterials, electrospun fibers have attracted attention due to their resemblance to collagen fibers in the extracellular matrix of healthy skin, thus providing an additional support for cell migration and proliferation [6].

**Figure 1:**
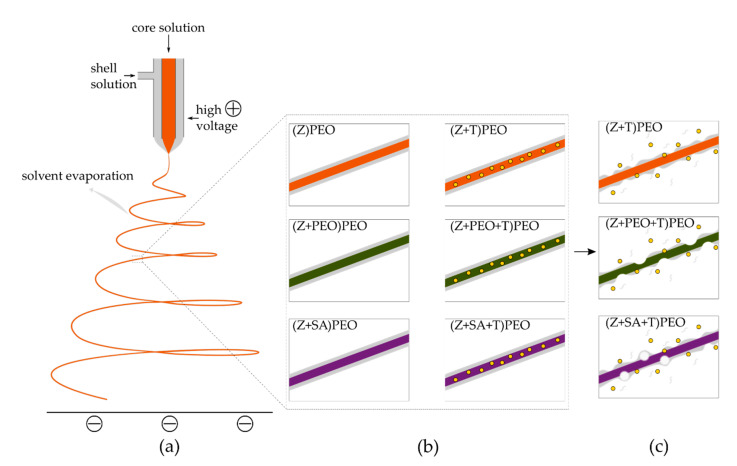
(**a**) Co-axial electrospinning set up with the core and the shell polymer solutions generating a spinning jet under a high voltage, from which the solvent is evaporated and dry fibers are collected on the oppositely charged plate. (**b**) Schematic representation of the developed fiber types, where the polyethylene oxide (PEO) shell is shown in light grey and the composition of the core is differentiated by the following colors: orange for zein (Z) only, green for zein with PEO, and violet for zein with stearic acid (SA). The drug tetracycline hydrochloride (T) is highlighted by yellow spheres. (**c**) Proposed drug release processes from T-loaded fibers. The process involves dissolution of the PEO shell in all fiber types and the burst release of dissolved T. Formation of pores during drug release was mostly observed in plasticizers-containing fibers. Larger pores were formed in (Z+SA+T)PEO. Nomenclature of the samples: composition of the core is shown in brackets, while the shell polymer is specified outside of the brackets.

Electrospinning is a process based on applying a high electric voltage to, for instance, a polymer solution. Under appropriate conditions (e.g., temperature, humidity level, flow rate and voltage), the high voltage transforms a drop of the polymer solution into a cone, known as a Taylor cone, in turn leading to the formation of a jet. The jet undergoes different bending instabilities resulting in spinning, during which solvent evaporates and dry fibers are deposited on the oppositely charged collector [7,8]. In the present study, a co-axial electrospinning approach was used. This involves the use of two polymer solutions, with the inner (core) solution being engulfed by the outer (shell) solution [9] (Figure 1a). Electrospinning polymers often requires the use of toxic organic solvents and cross-linking agents, to dissolve polymers and stabilize fibers, respectively. This poses a potential hazard to the environment and the manufacturer during the development process, as well as to the patient, if residual toxic substances remain in the final product [10]. To overcome these drawbacks, zein, a non-toxic and biodegradable protein derived from corn and generally recognized as safe, was selected for this study. Due to its amphiphilic nature, zein protein is able to self-assemble [11]. This property has led to its wide use as a drug delivery system in the form of films, gels, coating material, vaccines, particles and fibers [12]. Zein protein and peptides have also demonstrated anti-oxidant activity and resistance to microbial contamination [13,14]. Other advantages of zein include its high availability and low cost, due to its low nutritive value caused by the lack of key amino acids [12]. In comparison to other polymers, zein can be dissolved in an aqueous ethanol solution, and form fibers without any cross-linking agents [11,15,16].

However, electrospinning of zein in aqueous ethanol comes with a number of drawbacks, such as poor electrospinnability [17], ribbon-shape morphology [15,18], and low water stability [19,20], as well as brittleness and poor elasticity of the produced fibers [21,22]. Poor electrospinnability is mainly the result of rapid ethanol evaporation from the zein solution, which leads to an increase in viscosity and consequent clogging of the needle tip within seconds, thereby preventing the formation of the Taylor cone and the jet. This problem can be overcome by the use of co-axial electrospinning with aqueous ethanol as the shell solution [17,23]. Usually, zein fibers adopt the shape of flat ribbons instead of a classic tubular shape [15,18,21,24,25,26]. This is the result of the fast evaporation of ethanol from the surface, leading to the formation of a skin around the fibers, which collapses when the solvent within the fibers evaporates completely [15,27]. However, it has been shown that cells prefer tubular-shaped fibers for proliferation [28]. Researchers have therefore attempted to obtain tubular-shaped fibers by dissolving zein in organic solvents such as glacial acetic acid [29], or through the addition of polyethylene oxide (PEO) directly to zein in aqueous ethanol solution, followed by aging the mixture for several days to enhance hydration of its interior [30]. In another study, diluted acetic acid was employed and found to result in fibers only at low water concentrations, which showed a compact and dense structure [31]. Attempts to electrospin zein in a mixture of acetic acid, ethanol and water resulted in fibers with a beads-on-fiber morphology [32]. In addition to the challenges of electrospinning zein, the fabricated fibers tend to lose their porous structure and turn into a film upon contact with water [19,20,24,33]. Cross-linking with more toxic agents, such as formaldehyde [34], glutaraldehyde [35] and glyoxal [36], as well as less toxic ones such as citric acid [20,37], or by ultraviolet (UV) exposure [19], have been described as options to improve the stability of zein fibers in water. However, the use of citric acid requires 48 h for cross-linking of the solution, and high temperatures for further cross-linking of the fibers. Long UV exposure also poses a problem, as it may lead to a change in the chemical structure of polymers [38] or degradation of incorporated drugs [39].

Another drawback of zein fiber mats is that they demonstrate weak mechanical properties [21,22], which may not be desirable for a potential wound dressing that should ideally mimic skin elasticity [40]. Plasticizers may improve the electrospinnability of zein and the mechanical properties of zein fibers. PEO and different fatty acids, for example, have been reported to successfully plasticize zein films and increase their flexibility and elasticity [41,42]. PEO has further been shown to improve the electrospinnability of zein [43] and to increase the hydrophilicity of the fibers [44]. In the present study, we therefore chose PEO as both a plasticizer and shell-forming component to core–shell zein-based fibers. In addition, stearic acid (SA) was included in the core as plasticizer for zein, and as a skin-friendly component due to its presence as a free fatty acid in the stratum corneum [45]. Similarly, PEO is a non- toxic polymer, which is well tolerated in wound healing applications [46]. There is only a limited number of studies that produced fibers from zein and PEO by electrospinning, and to the best of our knowledge there are no studies on electrospinning of mixtures of zein and SA. With respect to studies focusing on zein and PEO, zein was uniaxially electrospun with PEO [30,32], and co-axially electrospun with zein as a shell and PEO as a core [47]. However, in all cases high PEO concentrations and low zein concentrations were required, to improve the electrospinning and prevent ribbon morphology from occurring. In our work, we aimed to improve the electrospinnability of zein, as well as the morphological and physico-chemical characteristics of zein fibers, by developing different types of core–shell zein-based microfibers and using small amounts of PEO or SA. The fibers were characterized and compared in terms of morphology, water vapor sorption, mechanical strength and elongation, and drug encapsulation and release, as well as cell safety.

## 2. Materials and Methods

### 2.1. Materials

All materials were purchased from Sigma-Aldrich (Sigma-Aldrich Inc., Darmstadt, Germany) unless specified otherwise. PEO (Mv ~900,000), SA (synthesis grade), zein (19~22 kDa), and tetracycline hydrochloride (T, ≥95% purity) were used to fabricate zein fibers. Antibiotics used for growth media preparation (penicillin, streptomycin), Glasgow Minimal Essential Medium (GMEM) (PAN Biotech, GMBH, Aidenbach, Germany), fetal bovine serum (FBS), tryptose phosphate broth (TPB, Difco), 4-(2-hydroxyethyl)-1-piperazineethanesulfonic acid) (HEPES), Dulbecco’s Modified Eagle medium (DMEM, phenol red and serum free medium), Lennox lysogeny broth (LB) and all other materials were of reagent grade, and were used as received without any further purification. Baby hamster kidney cells (BHK-21) were used for cell viability studies. Clinically relevant for wound infection, facultative anaerobic Gram-positive [*Staphylococcus aureus* (*S. aureus*) DSM No.: 2569, wound isolate] and Gram-negative bacterial strains [*Escherichia coli* (*E. coli*) DSM No.: 1103, clinical isolate] used in this study were obtained from the Leibniz Institute DSMZ-German Collection of Microorganisms and Cell Cultures (Braunschweig, Germany). Absolute ethanol was from VWR International (VWR as part of Avator, Søborg, Denmark), and milli-Q water was supplied by a PURElabflex dispenser (Elga-Veolia, Lane End, UK).

### 2.2. Preparation of Microfiber Mats

For the core solutions, all materials were dissolved in aqueous ethanol solution (1:4 *v/v*) at 60 °C. Either PEO or SA were added to some samples at 1% *w/w* of zein concentration and dissolved overnight. Then, 20% *w/v* zein was added 2 h before electrospinning to avoid aging. For drug-loaded fibers, T of 5% *w/w* of zein concentration was added to zein solution and allowed to mix for 10 min. The shell solution consisted of either absolute ethanol or 1% *w/v* PEO in aqueous ethanol (1:4 *v/v*). The electrospinner (Fluidnatek LE-50, Bionicia, Valencia, Spain) was set to 25 °C and 45% relative humidity (RH) throughout, whereas the other settings were chosen according to the material composition (see Table 1). All fabricated mats were stored at −4 °C at 0% RH before tests.

### 2.3. Surface Morphology

Samples were coated with gold (Sputter coater Cressington 108 auto, Ted Pella Inc., Redding, CA, USA) for 30 s and analyzed by scanning electron microscopy (SEM) on a TM3030 (Hitachi, Tokyo, Japan). Tests were conducted in triplicate. All images were analyzed with ImageJ software, DiameterJ plugin (1.52a version) [48]. At least 100 fibers were included for the fiber diameter and size distribution measurements for each type of the fibers.

### 2.4. Wettability

Wettability of the samples was analyzed using a Drop Shape Analyzer (DSA100, Krüss, Hamburg, Germany) at RT. Samples were cut into 10 mm^2^ pieces and the water contact angle measured using the sessile drop method. Measurements were conducted in triplicate.

### 2.5. Water Vapor Sorption

Water vapor sorption and desorption profiles were determined using a vapor sorption analyzer (VTI-SA+, TA instruments, New Castle, DE, USA). Samples were dried at 60 °C at a heating rate of 2 °C min^−1^ at 0% RH and then subjected to gradual increase in RH up to 90% at a constant temperature of 25 °C.

### 2.6. Water Stability

Fiber mats were cut to 6 mm diameter circles and sterilized on both side for 2 h with UV light (UVP 3UV lamp, Analytik Jena, Jena, Germany) at 245 nm [49]. The samples were immersed in phosphate-buffered saline (PBS) at pH 7.4 at 37 °C. After 48 h of incubation, the samples were removed, rinsed twice with milli-Q water and blotted with tissue paper. All samples were dried at 0% RH overnight and analyzed using SEM. Measurements were conducted in triplicate.

### 2.7. Solid State Characterization

Solid state characterization of the electrospun fiber mats and their starting materials was performed using differential scanning calorimetry (DSC) and X-ray diffraction (XRD). Thermal properties were determined using DSC on a TA Discovery (New Castle, DE, USA), and all samples were placed in hermetic pans with pierced lids. A heating rate of 2 °C min^−1^ was applied from −10 °C to 230 °C and modulated with a 0.3180 °C amplitude every 60 s. Measurements were conducted in duplicate. XRD patterns were obtained using an X’pert PRO (PANanalytical, Malvern, UK). All samples were scanned in the range of 10°–35° (2*θ*) at 45 kV and 40 mA every month for 4 months. Measurements were conducted in triplicate.

### 2.8. Mechanical Characterization

Tensile tests of dry and wetted electrospun fiber mat samples was conducted on a texture analyzer TA.XT plus (Stable Micro Systems, Godalming, UK). The thickness of the samples was measured prior to the test with a digital caliper (precision up to 0.001 mm, DML, UK). All samples were tested at an applied force of 0.01 N and a speed of 0.5 mm s^−1^ at RT. Dry samples were cut into 40 × 10 mm (length × width) pieces and the ends were covered with aluminum foil to prevent a premature break. Wet samples were placed on a plastic frame for extra support and immersed in PBS at 37 °C with pH 7.4 for 2 min and then gently blotted to remove excess water. The vertical sides of the frames were cut before the experiment. Measurements were conducted in quintuplicate.

### 2.9. Drug Loading and Release

Samples were cut into 6 mm diameter circles and sterilized with UV light as described in Section 2.6. Drug-free zein fibers were used as control. For drug release experiments, sterilized samples were placed into 24 well plates with milli-Q water at 37 °C at 200 rpm (neoMix thermomixer, neoLab, Heidelberg, Germany). At specific time points (0 min, 5 min, 10 min, 30 min, 1 h, 3 h, 14 days, 30 days), 1 mL aliquots were removed and replaced with fresh milli-Q water. The drug loading of the mats was determined by dissolving the mats in 80% *v/v* aqueous ethanol followed by sonication (UP50H, Hielscher Ultrasound Technology, Teltow, Germany) for 30 s at 80% amplitude, 0.8 cycle. The samples were then analyzed with high performance liquid chromatography (HPLC; Shimadzu, CTO-20A/20AC, Kyoto, Japan) at 300 nm using a ShimPack GIST C18 column (Shimadzu, Kyoto, Japan). Mobile phase A consisted of 10 mmol L^−1^ oxalic acid and mobile phase B consisted of 10 mmol L^−1^ oxalic acid and acetonitrile (1:1 *v/v*). The gradient was 18–60% B from 0 and 3.5 min and 60–18% B from 3.6 and 7 min. The column temperature was set to 40 °C and the flow rate to 1 mL min^−1^.

### 2.10. Agar Diffusion Assay

Samples were cut and sterilized as described in Section 2.6 and bacterial susceptibility measured using an agar diffusion method described previously [50]. *S. aureus* and *E. coli* were selected as model Gram-positive and Gram-negative bacteria, respectively. Briefly, overnight bacterial cultures in LB (grown from DMSO stocks) were seeded at 1 × 10^4^ colony forming units (CFU mL^−1^) after which samples of 6 mm diameter circles were placed on top together with controls and allowed to incubate in aerobic conditions for 24 h at 37 °C. Inhibition zones were measured using ImageJ software (1.52a version) [48]. The test was conducted in triplicate.

### 2.11. Cell Safety

The mats were cut and sterilized as described in Section 2.6. Cell viability in the presence of electrospun fiber mats was analyzed by a direct method described previously [50]. Briefly, fiber mats were placed into 24 well plates seeded with baby hamster kidney cells (BHK-21) at 5 × 10^4^ cells per well. Fiber mats without T, cells with growth medium without mats, cells seeded with T solution corresponding to the concentration in the fiber mats and cells with 0.1% Triton X-100 were used as controls. After 24 h of incubation at 37 °C with 5% CO_2_ supply, live and dead cells were counted using the trypan blue exclusion assay on an automated cell counter (Invitrogen, Thermo Fisher, Waltham, MA, USA). The viability was calculated (%) from live and dead cell numbers. The tests were conducted in triplicate.

Additionally, UV sterilized fiber mats were cut into 90 mm diameter circles and secured on the cell-crown inserts (CellCrown^®^, Scaffdex Oy, Tampere, Finland), which were placed into 24 well plates and seeded at 5 × 10^4^ cells per well. Cells were incubated as described above. After 24 h fiber mats were removed, cells were fixed with 4% formaldehyde for 30 min at RT and then dried at 0% RH for at least 2 weeks before analysis with SEM.

### 2.12. Statistical Analysis

All data were analyzed using Origin software (version 9.6.0.172, OriginLab Corporation, Northampton, MA, USA) and represented as means ± standard deviation (SD) with *p* < 0.001 considered significantly different. Kruskal–Wallis analysis of variance was performed to analyze the effect of the fiber composition and drug presence on the diameter and size distribution of the fibers and the DSC data. One-way analysis of variance followed by Tukey’s test for means comparison and Levene’s test for equal variances was applied to the rest of the data.

## 3. Results

### 3.1. Fiber Diameter, Surface Morphology and Electrospinnability

**Figure 2:**
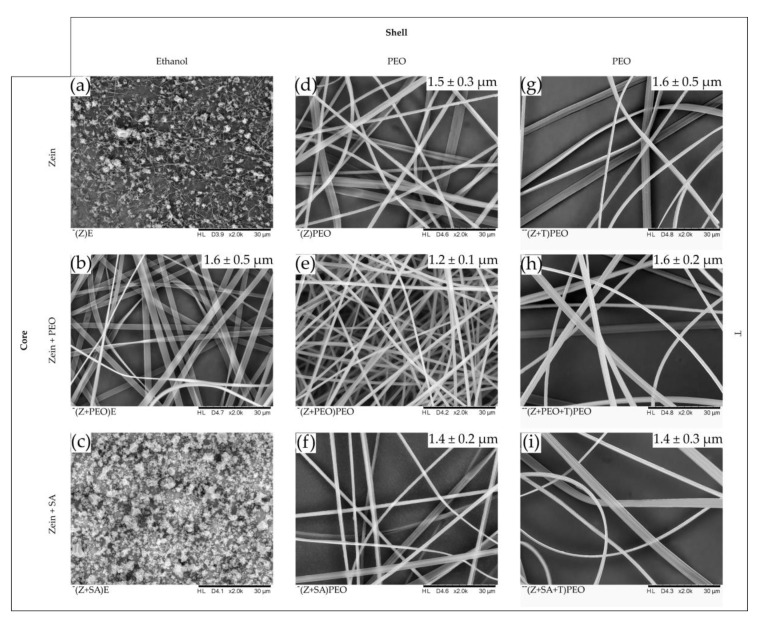
SEM images of the drug-free and drug-loaded zein (Z) fibers, magnification ×2000. Compositions of the core and shell solutions are indicated on the vertical and horizontal panes, respectively. The mean diameter size with standard deviation of the fibers is given in the white box in the upper right corner. (**a**,**c**) No fibers were produced when absolute ethanol (E) was used as shell solution with zein or zein + stearic acid (SA) as a core. (**b**) Fibers were produced with E as a shell solution only when polyethylene oxide (PEO) was present in the core, sample (Z+PEO)E. (**d**–**f**) PEO coated fibers. Tubular fibers were obtained when PEO was present both in the core and in the shell as in sample (Z+PEO)PEO. (**g**–**i**) Tetracycline hydrochloride (T)-loaded fibers. Incorporation of the drug changed the morphology towards more ribbon-shaped fibers, which led to an increase in the diameter distribution. Nomenclature of the samples: composition of the core is shown in brackets, while the shell polymer or solvent is specified outside of the brackets.

The diameters of the different fiber types were relatively similar and above 1 µm (see Table 2). However, the diameter distribution of the fibers was larger for samples that contained ribbon-shaped fibers compared to mostly tubular-shaped fibers, such as those formed in (Z+PEO)PEO. The morphology of the developed zein fibers was dependent on both PEO content and its location within the fibers. With PEO present only in the shell and SA in the core, the fibers exhibited mixed morphology of tubular and ribbon shapes (Table 2, Figure 2). A primarily tubular morphology of zein fibers was achieved when PEO was present both in the core and the shell. Beads were formed in samples, in which the core contained only zein or zein and SA, while the shell solution contained no PEO and instead only absolute ethanol, i.e., (Z)E and (Z+SA)E. The latter samples also displayed poor electrospinnability, characterized by an unstable jet and needle clogging, confirming the importance of PEO for improving zein electrospinnability. Therefore, they were excluded from further tests. Samples with zein and PEO in the core and ethanol in the shell solution, (Z+PEO)E, formed ribbon-shaped fibers and displayed continuous electrospinnability. Addition of T slightly changed the morphology of all fiber types, introducing a more ribbon-shaped morphology, which increased the diameter distribution of the fibers.

### 3.2. Wettability

The wettability of the fiber mats was analyzed by water contact angle measurements to determine the effect of the additives on the hydrophilicity of zein fibers. Results are summarized in Table 2. Even though (Z+PEO)E and (Z+SA+T)PEO displayed contact angles of approximately 100°, the water droplet was immediately absorbed by all samples within 1–3 s.

### 3.3. Water Vapor Sorption

The vapor sorption profiles of all fiber mats were relatively similar (Table 2). With increasing RH, the vapor sorption capacity reached approximately 21–26% in all samples, except for (Z+P)E that had a considerably lower vapor sorption of approximately 8%.

### 3.4. Water Stability

**Figure 3:**
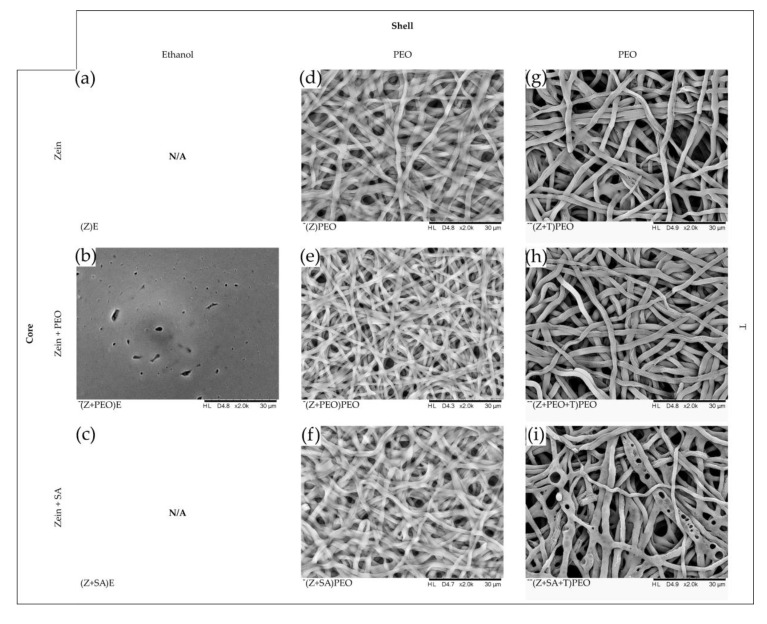
SEM images of zein fibers after 48 h of incubation in PBS, magnification of ×2000. Composition of the core and the shell solutions are indicated on the vertical and horizontal panes, respectively. (**a**,**c**) (Z)E and (Z+SA)E are excluded due to poor electrospinnability and formation of beads. (**b**) (Z+PEO)E has completely lost its fibrous structure and turned into a film. (**d**–**i**) All fibers coated with PEO demonstrate enhanced water stability. (**g**–**i**) Formation of differently sized pores was observed in the drug-loaded fibers. Key: E, absolute ethanol; PEO, polyethylene oxide; SA, stearic acid; T, tetracycline hydrochloride; Z, zein; N/A, not available. Nomenclature of the samples: composition of the core is shown in brackets, while the shell polymer or solvent is specified outside of the brackets.

The ability of fibers to keep their shape upon contact with water without losing the porosity of the fiber mat was analyzed by SEM after 48 h of incubation (Figure 3). Fiber mats without PEO shell lost their porosity and turned into a film (Figure 3b). Therefore, due to its low water stability, (Z+PEO)E was excluded from further analysis. All fibers with PEO shell were swollen, but retained their structure, demonstrating stability. Additionally, SEM analysis of the drug-loaded fiber mats revealed different degradation profiles depending on the fiber composition. Formation of small pores was observed in fibers with PEO in the shell (Z+T)PEO. More frequent occurrence of small pores was observed in (Z+PEO+T)PEO. Much larger pores were formed in (Z+SA+T)PEO. In contrast, there were no pores present in the drug-free fibers.

### 3.5. Solid State Characterization

**Figure 4:**
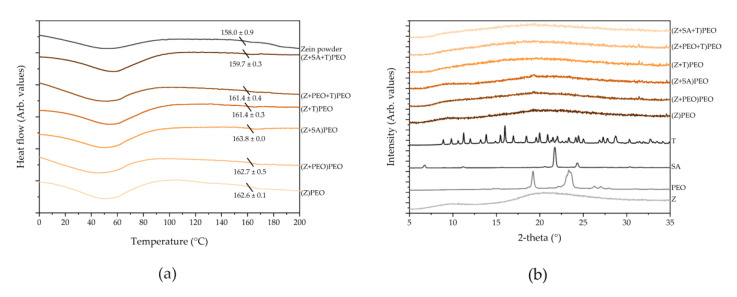
Solid state analysis of zein fibers. (**a**) Differential scanning calorimetry (DSC) thermograms showing the total heat flow. The diagonal line represents a mean glass transition temperature (Tg), which is given with standard deviation. (**b**) X-ray diffraction (XRD) patterns of fibers after 4 months (orange colors) and the raw powder components of the fibers (grey colors). Key: PEO, polyethylene oxide; SA, stearic acid; T, tetracycline hydrochloride; Z, zein.

**Figure A1:**
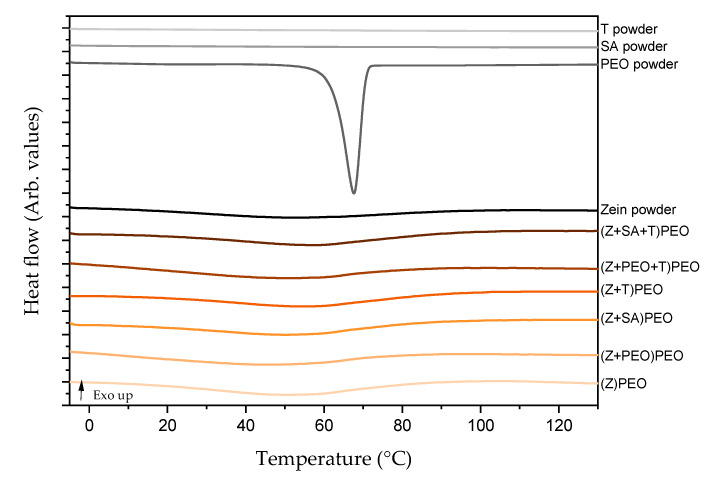
DSC thermograms of drug-loaded and drug-free zein fibers (orange color) and their raw powder forms that were measured on the day of preparation (black color). Key: PEO, polyethylene oxide; SA, stearic acid; T, tetracycline hydrochloride; Z, zein.

**Figure A2:**
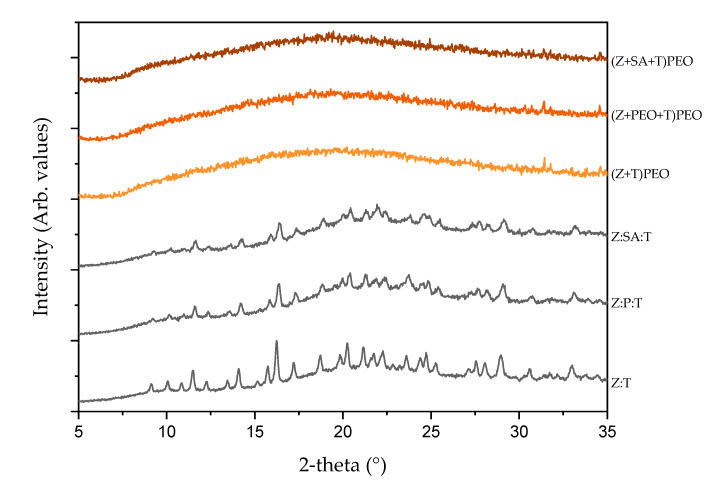
XRD pattern of drug-loaded zein fibers (orange color) and the corresponding physical mixtures with the same composition that were measured on the day of preparation (black color). Key: PEO, polyethylene oxide; SA, stearic acid; T, tetracycline hydrochloride; Z, zein.

DSC thermograms showed an endothermic peak below 100 °C in all fiber mats that corresponded to the evaporation of water [51] (Figure 4a and Figure A1). Zein is an amorphous material, and the glass transition temperature (Tg) for zein powder was 158.0 ± 0.9 °C, and for zein within fiber mats around 161.9 ± 1.3 °C (Figure 4a). Similar results were previously reported by Torres-Giner et al. [52,53]. Incorporation of T possibly decreased the Tg of zein slightly, however, this was not statistically significant. XRD patterns confirmed the presence of stable amorphous forms of all fiber components on the day of preparation (Figure A2), over the period of 4 months (Figure 4b), including T-containing samples over the period of 4 months.

### 3.6. Mechanical Characterization

**Figure 5:**
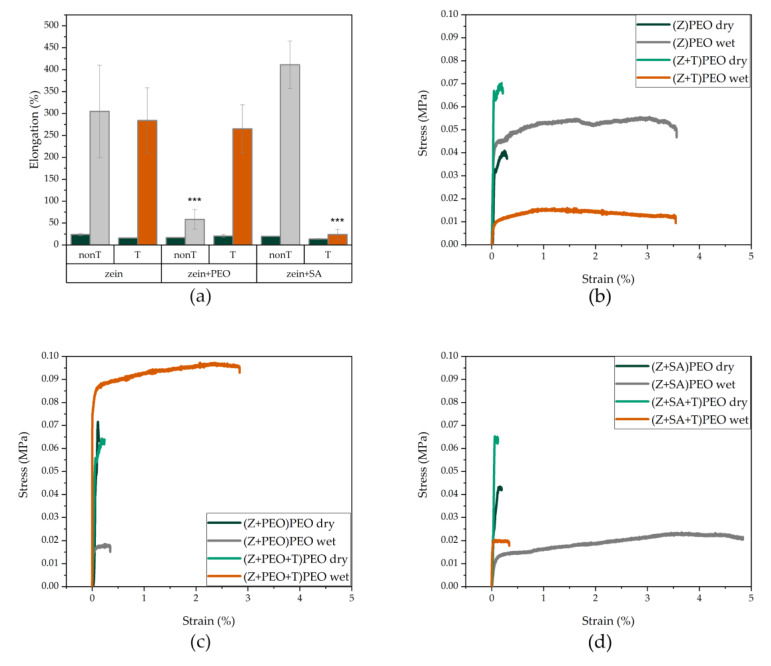
Mechanical properties of the dry and wet zein fibers. (**a**) Elongation at break, of dry fibers with and without tetracycline hydrochloride (T) in dark green, wet drug-free fibers in grey, and wet drug-loaded fibers in orange. (**b**–**d**) Stress-strain curves, of dry drug-free fibers in dark green, dry drug-loaded fibers in light green, wet drug-free fibers in grey, and wet drug-loaded fibers in orange. Key: PEO, polyethylene oxide; SA, stearic acid; T, tetracycline hydrochloride; Z, zein; error bars represent standard deviation; ***, statistically significant difference at *p* < 0.001.

To determine the strength, rigidity and elongation of the fabricated fiber mats, standard tensile tests were conducted (Table 3). Young’s moduli and tensile strengths in all samples were relatively low. However, elongation at break was considerably larger for wetted than for dry fibers [54]. Addition of PEO in the shell did not result in significant differences among the samples. On the other hand, stress-strain curves and elongation profiles of the wetted fiber mats were significantly affected by the composition of the fibers (Figure 5). Presence of PEO in the shell dramatically increased elongation, while PEO in the core significantly decreased elongation of fibers in the drug-free fibers (Figure 5a). Interestingly, addition of T to (Z+PEO)PEO increased elongation of these fibers (Figure 5a). The presence of SA in the core lead to an opposite elongation profile as compared to (Z+PEO)PEO, and the addition of T dramatically decreased the fiber elongation (Figure 5a).

### 3.7. Drug Loading and Release

**Figure 6:**
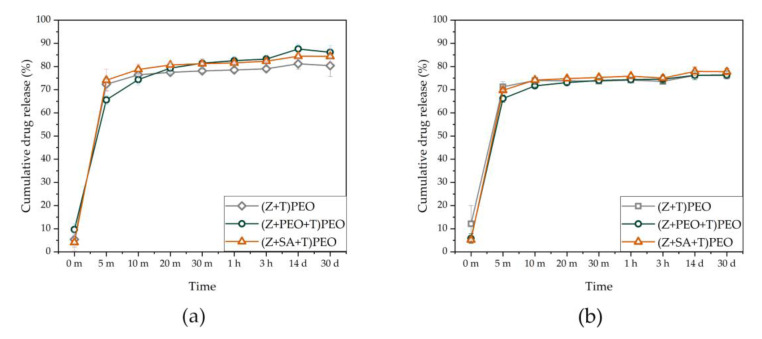
Drug release from zein fibers. (**a**) Cumulative drug release of tetracycline hydrochloride (T) and 4-epitetracycline from the freshly prepared samples. (**b**) Cumulative drug release of T and 4-epitetracycline from samples stored for 4 months. Key: PEO, polyethylene oxide; SA, stearic acid; T, tetracycline hydrochloride; Z, zein; m, minutes; h, hours; d, days; error bars represent standard deviation.

The encapsulation efficiency of T was high and relatively similar in all samples (Table 4). All fiber mats demonstrated burst release of T (Figure 6). There was a slight increase in the concentration of 4-epitetracyline, a degradation product of T [55,56], after 4 months of storage of the fibers (data not shown). Higher degradation of T was observed in PEO-containing fibers, which could be due to the hygroscopic nature of PEO.

### 3.8. Agar Diffusion Assay

**Figure A3:**
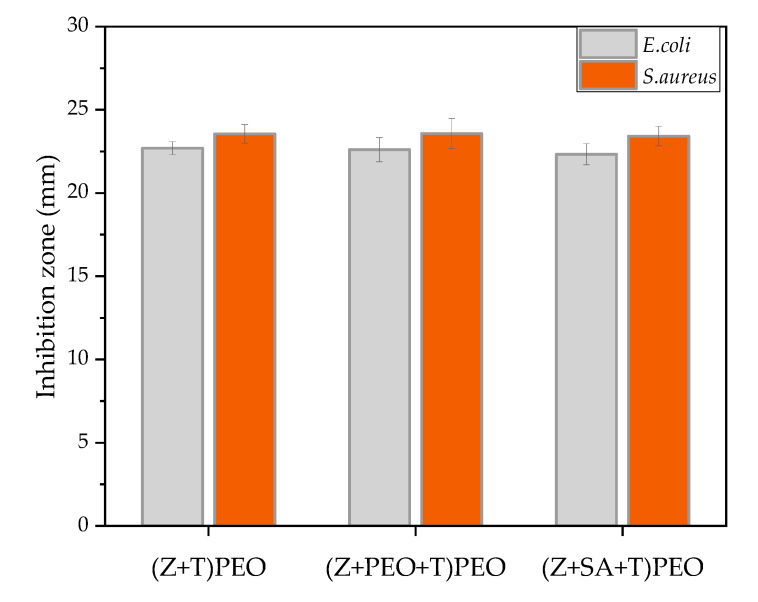
Antimicrobial activity of drug-loaded zein fibers against *E. coli* and *S. aureus* shown as diameters of inhibition zones from agar plates. PEO, polyethylene oxide; SA, stearic acid; T, tetracycline hydrochloride; Z, zein; error bars represent standard deviations.

To determine the antimicrobial activity of the T-loaded zein fibers, an agar diffusion assay was carried out with *S. aureus* and *E. coli*. These bacteria are known to be relevant pathogens for the development of wound infections [57], and are sensitive towards T [58,59]. After 24 h of incubation, the inhibition zones were approximately 23 mm for all samples, with no difference between the two bacterial species (Figure A3).

### 3.9. Cell Safety

**Figure 7:**
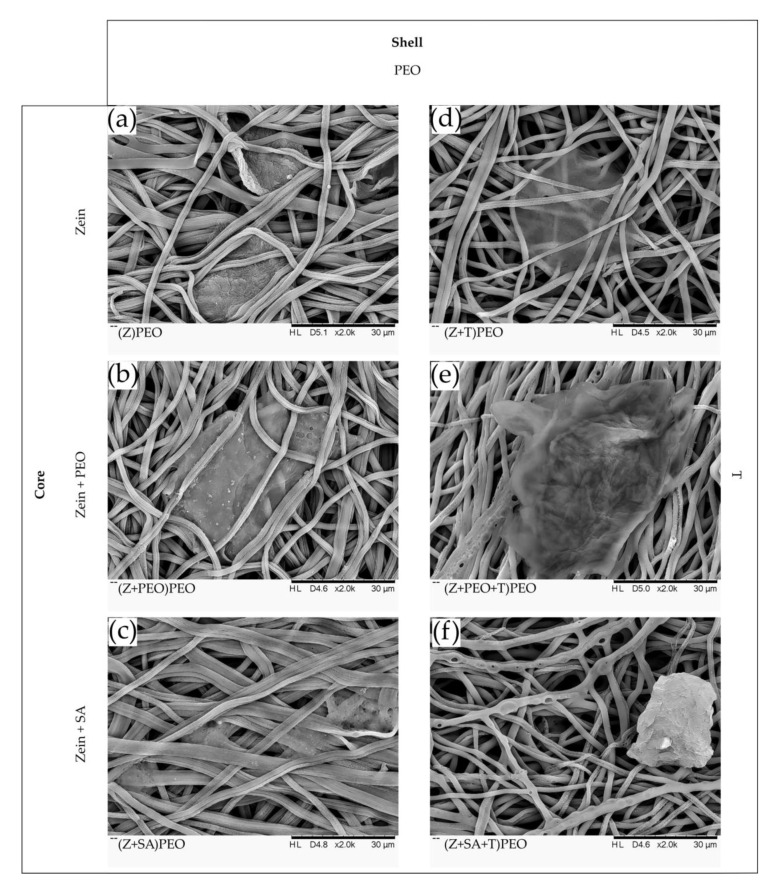
SEM images of zein fiber mats after 24 h incubation when seeded with kidney hamster fibroblast cells, magnification of ×2000. Composition of the core and the shell solutions are indicated on the vertical and horizontal panes, respectively. (**a**–**c**) Cells were mostly found entangled between the fibers in the drug-free samples. (**d**–**f**) Drug-loaded fibers. Cells were mostly present on the surface of the fiber mats containing plasticizers, which could be due to the formation of the larger pores along the fiber length that created a rough surface. Key: PEO, polyethylene oxide; SA, stearic acid; T, tetracycline hydrochloride; Z, zein. Nomenclature of the samples: composition of the core is shown in brackets, while the shell polymer is specified outside of the brackets.

**Figure A4:**
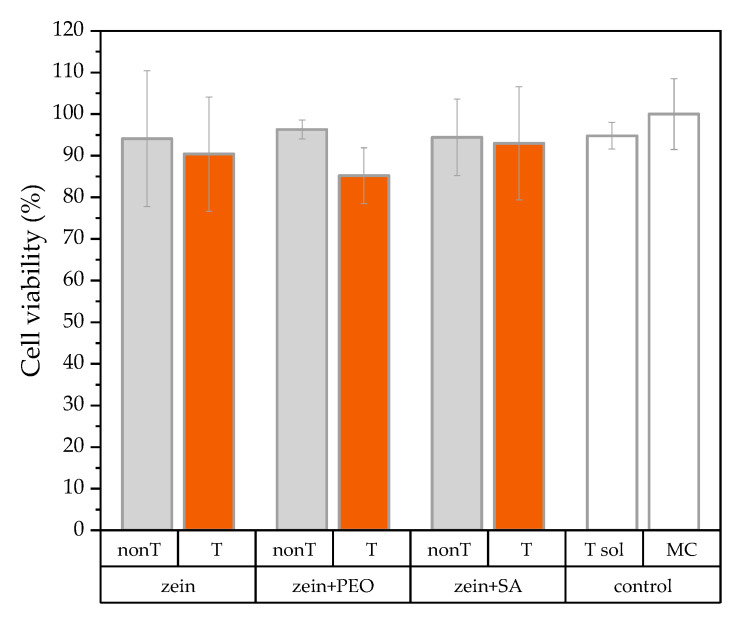
Cell safety results of drug-free (grey) and drug-loaded (orange) zein fibers on kidney hamster fibroblasts cells after 24 h of incubation. Results presented as relative cell viability (%). White bars represent controls of cells in T solution (T sol) and untreated cells in growth medium (MC). Key: PEO, polyethylene oxide; SA, stearic acid; T, tetracycline hydrochloride; nonT, no tetracycline hydrochloride; Z, zein; error bars represent standard deviations.

Cell safety was next investigated to obtain a first estimate of the biocompatibility of zein fiber mats. All samples demonstrated cell viability above 85% (Figure A4), with no statistically significant difference between the samples. Only slightly reduced cell viability was observed for drug-loaded zein fiber mats (Figure A4). SEM analysis revealed cell attachment on the fiber surface and cell migration within the pores of the mats (Figure 7). Clusters of cells were entangled between the fibers mostly in drug-free zein fibers and in sample (Z+T)PEO. However, most likely due to the presence of T and a change of fiber surface morphology (pore formation), cell behavior was altered, and cells were preferably placed on the surface of the fiber mats in (Z+PEO+T)PEO and (Z+SA+T)PEO.

## 4. Discussion

There is an increasing need for developing effective, well-tolerated and environmentally friendly biomaterials for wound healing. In this study, zein protein was electrospun co-axially with PEO in aqueous ethanol to obtain core–shell microfibers, with zein in the core and PEO in the shell. Further samples were prepared containing the plasticizers PEO or SA in the core together with zein. The main objectives included achieving continuous electrospinnability of zein protein, and introducing cell-friendly properties such as tubular morphology, hydrophilicity, elasticity and water stability.

The desired tubular-shape morphology of the zein fibers was achieved by the addition of PEO to both the core and shell solutions (Figure 2e). Presence of PEO only in the core or in the shell, respectively, resulted in the mixed morphology of tubular and ribbon-like fibers. This could be due to the dual effect exerted by PEO on zein in the core and in the shell. Hydrophobic interactions between PEO and zein have been described as changing the secondary structure of the protein by decreasing its alpha-helical content [60]. However, this interaction may have only occurred in the core solution that facilitated the formation of tubular-shaped fibers due to the unfolding of zein and its entanglement with PEO [61]. PEO in the shell, on the other hand, acted as a shield preventing immediate ethanol evaporation, and ensured a continuous electrospinning process.

Contact angle measurements showed the effect of PEO and SA on fabrication of zein fibers. Electrospinning zein with PEO considerably increases the hydrophilicity of the otherwise-hydrophobic zein microfibers. Despite the improved hydrophilicity, the water vapor sorption test demonstrated that the developed fibers only absorbed a limited amount of water vapor, which did not exceed 26% (Table 2). This possibly occurs due to the presence of a hydrophilic PEO shell that facilitates penetration of water molecules into zein, in comparison to the zein fibers without a PEO shell, (Z+PEO)E. However, the effect of the PEO shell is limited due to the amphiphilic nature of the zein protein.

Incorporation of T into zein fibers did not affect the wettability or vapor sorption of the fiber mats, but changed the morphology and mechanical properties of the fibers. In the presence of T, more ribbon-like fibers appeared in all samples, including (Z+PEO+T)PEO. This may be due to T reducing interactions between zein and PEO, which is consistent with the elongation profiles of (Z+PEO+T)PEO (Figure 5a). Young’s moduli and tensile strengths of all samples were lower than previously reported for zein fibers [21,22,26,54]. The achieved elasticity and low Young’s modulus corresponds to the data observed for skin [40]. Zein contains hydrophobic amino acid residues on its surface and is practically insoluble in water, yet water acts as a plasticizer [41], which explains the large elongation of zein fibers immersed in water for only 2 min. This large elongation has been shown before in wet plasticized zein films [41], but has not been reported for zein fibers before. The interaction of zein with PEO prevented fibers from undergoing larger elongation, as seen with (Z+PEO)PEO. However, addition of T to (Z+PEO)PEO, forming the sample (Z+PEO+T)PEO, displayed a reverse effect, most likely due to the release of T from the fibers, which resulted in the formation of small pores, allowing more water molecules to enter and dissolve PEO in the core. The exposure of zein hydrogen bonding sites, and their availability for interaction with water, led to an increased elongation of the fibers. In comparison, the high elongation profile of (Z+SA)PEO mats indicates that SA did not hamper hydrogen bonding between zein and water. High elongation of around 100% was reported for zein films plasticized with 12.5% SA and conditioned at high RH [62]. However, no information was found on either electrospinning zein with SA, or the mechanical properties of such systems. Addition of T dramatically decreased the elongation profile of (Z+SA+T)PEO, which was almost comparable to that of the dry fiber mats. This may be due to the formation of large pores, that weaken individual fibers and result in an early break of the fiber mats.

Despite the significant effect of the addition of small amounts of PEO and SA on the morphological and mechanical properties of zein fibers, the release of T from the developed systems was similar. The burst release most likely occurs due to the hydrophilic nature of T, its amorphous state within the fibers, and the coating with hydrophilic PEO, which increased water penetration into zein core through the formation of pores. This pore-generating property of polyethylene glycol has been previously reported for nanofibers with a poly(ε-caprolactone) core (PCL) and polyethylene glycol shell [63].

Even though T is known to have lower antimicrobial activity against *E. coli* than *S. aureus* [59], there was no statistically significant difference between the inhibition zones against either bacterial species. The susceptibility of the chosen *E. coli* strain could have affected the bacterial growth rate upon contact with T [58]. The drug concentration within zein fiber mats was 14.5 µg mL^−1^ for (Z+T)PEO, 13.7 µg mL^−1^ for (Z+PEO+T)PEO and 13.0 µg mL^−1^ for (Z+SA+T)PEO, which were 1.6–1.8 and 13–14.5 times above the minimum inhibitory concentration (MIC) for *E. coli* and *S. aureus*, respectively [64]. Hence, all T-loaded fibers efficiently released the drug and acted on bacteria. The viability of the fibroblasts in the presence of the developed zein microfibers loaded with T was more than 85%. The stability in water and the higher hydrophilicity of zein fibers with PEO in the shell, required for a potential application of these fiber mats in wound healing, facilitated cell attachment and migration, not only on the surface of the fiber mats, but also within the pores of the mats (Figure 7). However, this depended on either the changed morphology of the fibers (Figure 7) or the presence of T in the fibers [65], or a combination of both. Most often, cells are unable to penetrate into fiber mats due to the dense structure of the mats and small pore size, resulting in cell migration only on the surface of the mat. Nevertheless, a similar migration of cells inside zein/PCL-based fiber mats after 24 h of incubation has been shown before [66]. Based on the obtained data, the proposed mechanism of drug release involves dissolution of the PEO shell, which leads to the formation of pores along the fiber length, through which T is released and more water molecules are introduced into zein fibers (Figure 1c).

The presence of amorphous drug in zein fibers, and the high encapsulation efficiency, prolonged stability of the drug within the fibers, and its near-complete release from the fibers, demonstrates that the fabricated zein fibers may potentially be used as a fast release antimicrobial layer, that could be introduced into wound dressings to reach initial MIC. The fast release of antimicrobial agents is beneficial in heavily contaminated wounds, for eradicating existing bacteria and reducing the formation of a biofilm [67,68,69]. Successful optimization of the physico-mechanical properties of zein fibers, such as higher hydrophilicity, elasticity and better water stability, as compared to pure zein fibers, brings them closer to realizing the properties of an ideal wound dressing.

## 5. Conclusions

The present study reports on environmentally friendly and biocompatible, protein-based biomaterials for wound healing, showing elasticity and water stability without the use of any toxic solvents or cross-linking agents. The fabricated fibers were easily and continuously electrospinnable through the addition of PEO as a shell, resulting in a bead-free, tubular-shaped morphology. The obtained fibers showed higher hydrophilicity than zein fibers without PEO, as well as the ability to retain the porous structure of the mat upon contact with water. The addition of T to zein fibers led to a more ribbon-like morphology, and the loaded fibers were characterized by high stability of the drug over 4 months of storage. The high solubility and dissolution rate of amorphous T in fibers, and the hydrophilic nature of the fiber shell, led to a burst release of the drug. Drug-loaded and drug-free zein fibers displayed no toxicity against fibroblasts, and facilitated the migration of cells through the pores between the fibers, which makes them suitable for wound-healing applications.

## Figures and Tables

**Table 1:** Composition of the samples and the applied electrospinning settings.

Sample	Core	Shell	Flowrate (mL/h)		Voltage (kV)		Distance (cm)
			Core	Shell	Injector	Collector	
(Z)PEO	Zein	PEO	700	500	4.5	−0.6	18
(Z)E	Zein	EtOH	385	185	18.8	−2.7	23
(Z+T)PEO	Zein+T	PEO	700	500	4.5	−0.6	18
(Z+PEO)PEO	Zein+PEO	PEO	700	500	4.5	−0.6	18
(Z+PEO)E	Zein+PEO	EtOH	700	300	6	−1	18
(Z+PEO+T)PEO	Zein+PEO+T	PEO	700	500	4.5	−0.6	18
(Z+SA)PEO	Zein+Stearic acid	PEO	700	500	4.8	−1.8	23
(Z+SA)E	Zein+Stearic acid	EtOH	400	110	15.5	−3.5	13
(Z+SA+T)PEO	Zein+Stearic acid+T	PEO	700	500	4.8	−1.8	23

Key: EtOH, absolute ethanol; PEO, polyethylene oxide; T, tetracycline hydrochloride.

**Table 2:** Diameter, morphology, wettability and water vapor sorption of zein fibers.

Sample	Diameter (µm ± SD)	Morphology	Contact Angle (° ± SD)	Vapor Sorption (%)
(Z)PEO	1.5 ± 0.3	Tubular fibers	64.0 ± 15.4	26.5
(Z)E	N/A	Beads	N/A	N/A
(Z+T)PEO	1.6 ± 0.5	Tubular fibers	68.9 ± 16.7	23.1
(Z+PEO)PEO	1.2 ± 0.1	Tubular fibers	47.1 ± 4.7	24.3
(Z+PEO)E	1.6 ± 0.5	Ribbon fibers	110.6 ± 9.1 ***	8.2
(Z+PEO+T)PEO	1.6 ± 0.2	Tubular fibers	59.7 ± 17.6	21.5
(Z+SA)PEO	1.4 ± 0.2	Mixed fibers	72.1 ± 7.4	22.4
(Z+SA)E	N/A	Beads	N/A	N/A
(Z+SA+T)PEO	1.4 ± 0.3	Mixed fibers	100.6 ± 8.4 ***	24.3

Key: E, absolute ethanol; PEO, polyethylene oxide; SA, stearic acid; T, tetracycline hydrochloride; Z, zein; N/A, not available; SD, standard deviation; ***, statistical difference at *p* < 0.001.

**Table 3:** Mechanical properties of dry and wet zein fiber mats.

Sample	Young’s Modulus (MPa ± SD)		Tensile Strength at Break (kPa ± SD)		Elongation at Break (% ± SD)	
	Dry	Wet	Dry	Wet	Dry	Wet
(Z)PEO	1.5 ± 1.0	0.9 ± 0.4	54.6 ± 13.2	34.4 ± 17.1	22.7 ± 3.4	304.9 ± 105.8
(Z+T)PEO	4.3 ± 1.8	1.0 ± 0.9	85.1 ± 23.4	50.0 ± 47.6	16.7 ± 1.5	284.2 ± 74.7
(Z+PEO)PEO	4.2 ± 2.6	1.1 ± 0.4	75.2 ± 19.0	24.6 ± 7.1	17.4 ± 1.3	58.5± 22.4 ***
(Z+PEO+T)PEO	1.8 ± 0.9	0.5 ± 0.2	69.1 ± 20.5	44.2 ± 34.9	18.4 ± 4.1	265.1 ± 54.6
(Z+SA)PEO	1.6 ± 0.3	0.2 ± 0.1	58.0 ± 17.8	22.7 ± 5.3	19.3 ± 0.8	411.2 ± 54.3
(Z+SA+T)PEO	1.6 ± 1.0	1.2 ± 0.3	51.6 ± 12.9	18.7 ± 6.6	14.6 ± 1.7	24.0 ± 11.5 ***

Key: PEO, polyethylene oxide; SA, stearic acid; T, tetracycline hydrochloride; Z, zein; SD, standard deviation; ***, statistically significant difference at *p* < 0.001.

**Table 4:** Drug loading and encapsulation efficiency of T.

Sample	Drug Load (%)	TDL (%)	EE (% ± SD)	
			Fresh	4 Months
(Z+T)PEO	5	4.5	99.1 ± 6.2	89.6 ± 1.6
(Z+PEO+T)PEO	5	4.5	95.0 ± 1.4	93.0 ± 0.4
(Z+SA+T)PEO	5	4.5	89.0 ± 0.3	90.6 ± 0.7

Key: EE, encapsulation efficiency; PEO, polyethylene oxide; SA, stearic acid; T, tetracycline hydrochloride; TDL, theoretical drug load; Z, zein; SD, standard deviation.
